# Lower Airway Microbiota

**DOI:** 10.3389/fped.2019.00393

**Published:** 2019-09-27

**Authors:** Giulio Pulvirenti, Giuseppe Fabio Parisi, Alessandro Giallongo, Maria Papale, Sara Manti, Salvatore Savasta, Amelia Licari, Gian Luigi Marseglia, Salvatore Leonardi

**Affiliations:** ^1^Department of Clinical and Experimental Medicine, University of Catania, Catania, Italy; ^2^Unit of Pediatric Emergency, Department of Human Pathology of the Adult and Developmental Age “Gaetano Barresi”, University of Messina, Messina, Italy; ^3^Department of Pediatrics, Foundation IRCCS Policlinico San Matteo, University of Pavia, Pavia, Italy

**Keywords:** microbiota, microbiome, lung, airway, asthma, infections, immunity, probiotics

## Abstract

During the last several years, the interest in the role of microbiota in human health has grown significantly. For many years, the lung was considered a sterile environment, and only recently, with the use of more sophisticated techniques, has it been demonstrated that colonization by a complex population of microorganisms in lower airways also occurs in healthy subjects; a predominance of some species of Proteobacteria, Firmicutes, and Bacteroidetes phyla and with a peculiar composition in some disease conditions, such as asthma, have been noted. Lung microbiota derives mainly from the higher airways microbiota. Although we have some information about the role of gut microbiota in modulation of immune system, less it is known about the connection between lung microbiota and local and systemic immunity. There is a correlation between altered microbiota composition and some diseases or chronic states; however, despite this correlation, it has not been clearly demonstrated whether the lung microbiota dysbiosis could be a consequence or a cause of these diseases. We are far from a scientific approach to the therapeutic use of probiotics in airway diseases, but we are only at the starting point of a knowledge process in this fascinating field that could reveal important surprises, and randomized prospective studies in future could reveal more about the clinical possibilities for controlling lung microbiota. This review was aimed at updating the current knowledge in the field of airway microbiota.

## Introduction

The “microbiota” consists of different species of microorganisms that live in a defined environment. In our body, microbiota are present in organs that are in contact with the outside environment, mainly the gut, but now we know that microbiota is also present in lung. Microbiota differs among various individuals and also varies according to pathological events or the person's health state, and it can modulate immune responses. The term “microbiome” is considered to include the complete set of microorganisms (bacteria, viruses, and fungi) with their genomes (1). There are numerous mutually beneficial interactions between the human body and microbiota with metabolic reactions, which are important for our health and can contribute to the pathogenesis of some diseases (2). The first contact with bacteria seems to happen prenatally (*in utero*) via a microbial transfer at the fetomaternal interface at which point microbial DNA has been discovered (3). In fact the maternal–fetal unit is not sterile as was previously believed. Fetal colonization occurs through vertical transfer in the placenta of non-pathogenic commensal microbiota from the phyla Firmicutes, Tenericutes, Proteobacteria, Bacteroidetes, and Fusobacteria (4). During birth, babies have their first important contact with the external world and subsequently, begin to undergo colonization by external bacteria (5). There is a potential relationship between microbial composition that is formed during the initial time after birth for the early life environment and health outcomes, such as allergic diseases (6, 7).

The aim of this review was to understand the lung microbiota function and the interactions between pathogens and commensal microbes and to improve the possibility of prevention and treatment of airway infections in children. A futuristic and innovative project about prevention of pediatric airway infections was initiated in 2007 by the United States National Institutes of Health in order to better understand the microbial communities in our body and their role in health and disease (8).

## Methods

This review was conducted using two databases: (1) PubMed and (2) Science Direct. Using these websites, we searched for articles in English using the following key words: (1) airway microbiota; (2) lung microbiota; (3) asthma and microbiota; (4) infections and microbiota; (5) microbiota and immunity; and (6) probiotics in airway diseases. As a rule of thumb, we decided to use the abstracts of articles to assess whether the articles fit the topic. We also reviewed the references of the selected articles and read those with titles that might be of interest for the topic.

## Airway Microbiota

The lower airways were previously considered to be a sterile environment, but this has been demonstrated to be false, and recently, using culture-independent techniques, the presence of Actinobacteria, Proteobacteria, Bacteroidetes, and Firmicutes ribosomal DNA has been shown to exist in healthy people's lungs (9). Bacteria have been identified using sensitive identification techniques, including the 16S rRNA gene, which is specific for bacterial cells (10–12). Lung microbiota is composed of about of 2,000 bacterial genomes per cm^2^. Next generation sequencing (NGS) of 16S rRNA has allowed accurate identification of bacterial genetic material in the lung of healthy subjects, and a significant increase of Proteobacteria presence was found in asthmatic children (13).

The main contributor to the lower airway microbiota composition seems to be the upper airway microbiota (14). Direct contact between the upper and lower airway microbiota can happen more frequently than previously thought, also for aspiration which commonly occurs in healthy young subjects during sleep (15). Aspiration of oropharyngeal secretions, micro aspiration, or direct dispersal by contiguous mucosa creates the microbiome environment in the lung (16). Lung colonization starts immediately after birth (17), and the gut microbiota is established in the first years. Thereafter, it maintains this initial composition stability throughout life (18).

### Role of Environment

The microbiota is enriched by the environment, especially during childhood during which time specific immunity and tolerance to antigens are developed. A protective role against asthma and allergies emerges in children who grow up on a farm, showing the importance of exposing children to different microbes from animals and plants in the environment (19). Limited exposure to environmental bacteria and fungi from different conditions in life and overuse of antibiotics are responsible for the increase of autoimmune diseases in the last decades (20). Children who live in cities have less exposure to environmental microbes, and they present a higher incidence of allergy and asthma than children who were born in a rural environment, who have a lower probability of having asthma. There are different mechanisms of this protective role in inflammatory disease; one of them could be the activation of mucosal invariant natural killer T cell (iNKT) tolerance, which is caused by early contact with these antigens (21).

### The Gut/lung Axis

The metabolites produced by the gut microbiome in the intestinal microenvironment consist of short-chain fatty acid (SCFAs) that reach other organs and influence lung respiratory disease (22–24). There is clear evidence concerning the contribution to lung immunity by the gut microbiota in the gut–lung immunity axis, such as during pneumococcal pneumonia in which macrophage functions increase at alveolar sites (25). Dietary changes influence the composition of the gut microbiota and asthma and allergic disease statuses (26). The influence of nutrition, in particular dietary composition, on lung immunity was examined, and it was shown that a diet rich in fiber causes an increase in circulating SCFAs levels, which are produced by fiber fermentation in the gut. This fermentation process prevents allergy and asthma-related inflammation of the lower airway (27). Lung SCFAs are produced by gut bacteria, and these are the main metabolic products of anaerobic bacteria fermentation. SCFAs promote recruitment and activation of leukocytes and immune regulation within the inflammatory process (28), and B cell differentiation occurs through regulated gene expression that supports the antibody production (29, 30).

## Microbiota and Asthma

**Figure 1:**
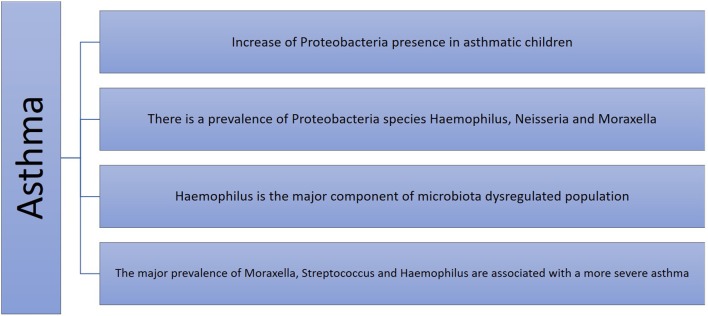
Association of microbiota dysbiosis in patients with asthma.

Asthma is the most frequent chronic respiratory disease in children, and its beginning and development is influenced in the first years of life by genetic predisposition and environmental factors, such as antibiotic exposure, breastfeeding, and contact with animals and the natural environment, which play an important role in asthma and other inflammatory disease development (31). In mice, the positive or negative influence of specific bacteria have been investigated with respect to detecting the susceptibility to allergic asthma (32). In humans, microbiota dysbiosis of some bacterial communities has been strongly associated with asthma (33–35). In fact, a difference in microbiota composition between asthmatic patients and healthy control subjects has been demonstrated. The former have a predominance of Lactobacillus, Pseudomonas, Rickettsiae, and Proteobacteria species, and the latter are characterized by population of *Prevotella, Streptococcus, Veillonella, Firmicutes*, and *Actinobacteria* (Figure 1). However, studies show different results according to sampling, technique isolation, and examined population (age, asthma severity, and control) (36–40). Patients with more severe airway obstruction and those who require higher doses of inhaled corticosteroids or oral corticosteroids have higher pathogenetic species than asthmatic patients with better-controlled disease (36). Species belonging to Proteobacteria phylum (*Comamonadaceae, Sphingomonadaceae*, and *Oxalobacteraceae*) are considered markers of worse disease control and are predictors of bronchial hyperresponsiveness (39, 40).

Lower airway microbiota in asthmatic patients is also related to a different upper airway microbiota composition. In fact, early alterations in oral microbial composition emerged in children who were developing asthma and who had a lower diversity of salivary bacteria together with highly divergent bacterial composition. These early changes seem to influence immune maturation and allergy development (41). Beyond the mouth, nasopharyngeal microbiome diversity can change over time in children with asthma; in particular, the relative proportions of *Haemophilus, Moraxella, Staphylococcus*, and *Corynebacterium* genus change over time (42). The results from a prospective cohort of 234 children show that children with early asymptomatic *Streptococcus* nasopharynx colonization in the first months of life have sensitization to allergens and a major risk of asthma development (43, 44). Also the nasal microbiota seems to be important; in fact, in children with asthma, the microbiome study from nasal secretion samples shows a distinct nasal airway microbiota-dominated Moraxella species associated with increased exacerbation risk (45, 46).

The asthma phenotype derives from an endotype that is based on the prevalence of a specific inflammatory cells and cytokines, which can be influenced by the microbiota (47). The neutrophilic phenotype, which is associated with a more severe clinical course and is refractory to corticosteroids (48), has been associated with a specific microbiota in the airway environment (49). An altered bacterial profile with more *Neisseria, Bacteroides*, and *Rothia* species was found in patients with low eosinophilic levels (50). The main prevalence of *Moraxella, Streptococcus*, and *Haemophilus* genera were associated with more severe asthma, and the phylum Proteobacteria was the most prevalent in Th17 cell-mediated asthma (51). Table 1 summarizes the main associations between microbiota and asthma.

**Table 1:** Main associations of microbiota in asthma.

**Type of study**	**Population**	**Technique of microbiota identification**	**Sampling**	**Microbiota results**	**Limitation**
Case-control (36)	39 asthmatic patients and 19 control subjects	16S rRNA V4 amplicon sequencing	Endobronchial brushings and bronchoalveolar lavage fluid	*Lactobacillus, Pseudomonas*, and Rickettsia in asthmatic patients. *Prevotella, Streptococcus*, and *Veillonella* species in control subjects	Limited number of subjects
Case-control (37)	10 non-asthmatic subjects and 10 patients with mild active asthma	DNA extracted from sputum supernatants and amplified by using primers specific for the V6 hypervariable region of bacterial 16S rRNA	Induced sputum	Proteobacteria in asthmatic patients *Firmicutes* and *Actinobacteria* in non-asthmatic subjects	Limited number of subjects
Case-control (39)	65 patients with suboptimally controlled asthma and 10 healthy control subjects	High-density 16S ribosomal RNA microarray and parallel clone library-sequencing analysis	Bronchial epithelial brushings	16S ribosomal RNA amplicon concentrations and bacterial diversity were significantly higher among asthmatic patients. Airway microbiota composition and diversity were significantly correlated with bronchial hyperresponsiveness	Pilot study
Case-control (40)	42 atopic asthmatic subjects, 21 subjects with atopy but no asthma, and 21 healthy control subjects	16S rRNA gene sequencing	Bronchial brushing	*Haemophilus, Neisseria, Fusobacterium*, and *Porphyromonas* in asthmatics	
Cohort study (41)	Children developing allergic symptoms and sensitization (*n* = 47) and children staying healthy (*n* = 33) up to 7 years of age	Illumina sequencing of the 16S rDNA gene	Oral bacterial composition in saliva samples collected at 3, 6, 12, and 24 months, and 7 years of age	Lower diversity of salivary bacteria and highly divergent bacterial composition at 7 years of age in children developing asthma	
Cohort study (42)	40 children and adolescents with asthma	Sequence data from the 16S-V4 rRNA gene region	Nasopharyngeal washes	*Moraxella, Staphylococcus, Dolosigranulum, Corynebacterium, Prevotella, Streptococcus, Haemophilus, Fusobacterium*, and a *Neisseriaceae* genus accounted for 86% of the total reads	
Prospective cohort study (43)	234 children	16S rRNA gene deep sequencing	Nasopharygeal samples	Early asymptomatic colonization with *Streptococcus* was a strong asthma predictor	
Cohort study (46)	413 Children with enrolled in a trial of omalizumab (anti-IgE)	16S rRNA profiling	Nasal secretion samples	*Moraxella* species were associated with increased exacerbation risk and eosinophil activation. *Staphylococcus* or *Corynebacterium* species-dominated microbiotas were associated with reduced respiratory illness and exacerbation events	
Multicenter randomized controlled trial (49)	167 adults	16S rRNA gene sequencing	Induced sputum specimens	A greater frequency of pathogenic taxa at high relative abundance and reduced *Streptococcus, Gemella*, and *Porphyromonas* taxa relative abundance in patients with neutrophilic asthma	

In patients with severe asthma, antibiotics can change the microbiota composition and also influence the clinical state; an example of such an antibiotic is azithromycin, which can reduce exacerbations and improve the quality of life in patients with uncontrolled asthma undergoing long-term treatment for their disease (52). Inflammation of the airway surface could be an important primary factor that changes the microbiota composition; however, the interdependent dynamics between the asthmatic host and the microbiota are not fully understood (53).

### Early Life Events and Asthma

The environment could be a risk or a protective factor, and the farming environment has been suggested to be protective against asthma and other allergic diseases that are likely affected by microorganisms, which influence the innate immunity and determine the early gene expression that will remain throughout life (54). Children who live in a rural environment, such as a farm, have a significantly reduced risk of asthma and atopic dermatitis (55). The decreased exposure to microorganisms during early life predispose children to develop asthma (56). The first year is a critical time for microbiota maturation, and an immature gut microbiota composition is linked to an increased risk of asthma at 5 years of age (57). For example, early colonization by *Clostridium difficile* at 1 month of age has been associated with later asthma or wheezing development (58). The use of some antibiotics, such as macrolides, in early life creates a compositional shift in the intestinal microbiota with depletion of *Actinobacteria* and an increase in Proteobacteria and Bacteroides. This shift has been correlated with an increased risk of immunological diseases, such as asthma, in children (59). The use of antibiotics in the prenatal period seems to be associated with an increased risk of asthma. Altered metabolic short-chain fatty acid (SFCA) levels were found in children who are at risk of developing asthma (60) and the mother's exposure to a rural environment during pregnancy with a connected increase to circulating SCFAs has also been associated with a decrease in the rate of asthma development in children with the possible induction of regulatory T lymphocytes in the fetal lung as was demonstrated in mice (61).

## Infections and Pathology

The healthy lung is colonized only by a limited population of bacteria that are maintained by an equilibrium among immigration, elimination, and growth. Some changes in the local environment during pathology can permit an increase in some bacterial populations that could became pathological, especially in chronic conditions (62). Lung microbiota could be involved in wheezing development because of the association between lower airway infections during the first years of life and an increase in the risk of wheezing. The number of episodes of airway infections in the first year of life has been associated with the later risk of developing asthma (63). Susceptibility to pulmonary infections depends on the stability of microbiota composition built in the first years as seen in the early life profiles that contain more *Moraxella* and *Corynebacterium/Dolosigranulum* in the upper respiratory tract of children (17). There is a direct effect of viral infections in the overgrowth of some pathogenic bacteria, such as a significant increase in the nasopharyngeal load of *Streptococcus pneumoniae* in children with influenza (64). After influenza A infection, there is an alteration in the microbiota equilibrium that results from direct physical mucosal damage. However, there are also changes in some host-produced immune modulating molecules or cytokines, which can alter the fine interaction between different colonizing bacteria and biofilm formation with their microenvironment. This permits pathogenic invasion as seen between *S. pneumoniae* and *Staphylococcus aureus* in the transition to secondary pneumonia (65). Moreover, in patients with influenza, there is an overgrowth of Proteobacteria, such as *Enterobacter* and *Moraxella* (66). Rhinovirus infection can influence the microbiota composition as demonstrated in chronic obstructive pulmonary disease patients in whom there is a significant growth in the *Haemophilus influenzae* population (67). Viral infections, such as respiratory syncytial virus (RSV) infection with a documented increase in *Moraxella* and *Haemophilus* members of the phylum Proteobacteria, alter the microbiome composition and influence the susceptibility to asthma (68).

The presence of a particular microbiota signature during upper respiratory tract infections in children compared to healthy patients shows that children with viral infections have a higher density and frequency of colonization with *S. pneumoniae, M. catarrhalis*, and *H. influenzae* in the nasopharynx (69).

Some changes in microbiota composition, such as a role for fungal microbiota in inflammation, have been observed in patients with chronic rhinosinusitis, allergy, cystic fibrosis (CF), and asthma (70). There is a crucial relationship between the airway microbiome and the stage and clinical progression of CF that is connected to the reduction in bacterial population diversity (71). In sputum samples from CF children, chronic colonization of specific pathogens, such as *Pseudomonas aeruginosa* was identified (72, 73), and in these patients it may be possible to use the microbiome as a primary target of prevention. In fact, a reduction in the exacerbation after the administration of the probiotics *Lactobacillus casei* and *L. rhamnosus* has been shown (74).

## Microbiota and Immunity

There are more bacterial (prokaryotic) cells than eukaryotic cells in our body. The cells are interconnected with the organs, and they form an important part in the regulation of immune system function (75). The microbiota is directly connected to the immune system, and it undergoes metabolic and antigenic interactions. The gut and lung are interconnected, and dysbiosis in the gut microbiota is also associated with lung diseases because the microbiota participates in the development and maintenance of the immune system. Because dysbiosis can permit disease development, immunity can also influence the microbiota composition, which provides resistance to colonization by respiratory pathogens that have a reciprocal influence on maturation and health maintenance (76). Regulation of the balance between Th-mediated inflammation and the T-regulatory response to environmental allergens can be controlled by skin commensals, such as *Acinetobacter* (77), and the same important role may be played by other microorganisms in the lung. The overgrowth of some species of lung microbiota with a reduction in species diversity could cause an inflammatory cell-mediated host response that is connected with alveolar tissue remodeling with consequent chronic changes (78).

The metabolites produced by bacteria can activate alveolar macrophages through nuclear factor kappa-light-chain-enhancer of activated B cells (NF-κB) and also activate the cell response through the Toll-like receptor (TLR) link, thereby influencing airway immunity function (79).

The creation of an airway microbiota within the first weeks of life is connected with early development of immune system tolerance and includes the induction of regulatory cells with specific genetic activation of innate immune cytokines (interleukins-4, -5, and -13) and cell ligands that are observed in mice (80). The microbe stimuli can then influence maturation of the lung mucosal barrier with homeostasis of the immune regulatory surface that is in contact with the environment, thus expanding the regulatory lung interstitial macrophages and influencing the susceptibility to allergic asthma (81). The segmented filamentous bacteria that are present in the gut microbiota induce autoantibodies in the lung through a Th17 T-cell-receptor-mediated inflammatory response (82).

For the possible role of immunity-related genes in microbiome composition, the role of genetic variations in mucosal immunity pathways on the upper airway microbiome has been investigated. Previous findings have shown an interesting association between the relative abundance of *Dermacoccus* and the variant 8 kb upstream of TINCR, a long non-coding RNA that binds to peptidoglycan recognition protein 3 (PGLYRP3) mRNA, which is a gene encoding a known antimicrobial protein. Moreover, the association between a missense variant in PGLYRP4 (rs3006458) and the relative abundance of an unclassified genus of family Micrococcaceae (phylum *Actinobacteria*) has also emerged (83).

## Probiotics

There is a growing interest in the potential future use of probiotics for promoting stronger immune lung function by manipulating microbiota interactions in airways health. The immune function derived by immune system homeostasis represents the mucosal barrier and its microbiome environment interactions in the intact barrier surface; when this unit is disrupted, immunity is compromised for a long time after an acute infection (84). Probiotics could play a role in promoting immune modulation in innate immune function, such as via recruitment of natural killer lymphocytes (85). The efficacy of the immune response in the lung seems to correlate with the microbiota composition and connect with metabolism products, which are derived from these living organisms. There are microbiota profiles that are distinct in composition and distinct from the upper airways with respect to composition and structure in the lower airways, and these are associated with different local immune responses and peripheral metabolic reprogramming (86). In infant mice, intranasal administration of the commensal *C. pseudodiphteriticum* was shown to provide a protective influence through innate immunity activation that was mediated by TLR-3 against RSV infection and secondary pneumococcal pneumonia infection (87). In a prospective pilot study of children with CF, the administration of probiotics, particularly *Lactobacillus* GG, was shown to cause a reduction in the pulmonary exacerbation rate (88). Four randomized trials, in which the administration of *L. Rhamnosus GG* in children was studied, reported a reduction in the incidence of acute otitis and upper respiratory infections (89). Probiotic administration, in particular *L. rhamnosus*, within the first 2 years of life, has been demonstrated to reduce the incidence of allergy in children (90). Probiotic supplementary therapy has been investigated in many randomized trials for investigating clinical efficacy in common respiratory childhood diseases, such as asthma, rhinitis, or wheezing. Singular randomized placebo-controlled studies sometimes have shown an interesting effect on asthma after the administration of probiotics, such as *L. paracasei* (LP) and *L. fermentum* (LF) (91). In two randomized double-blind placebo-controlled trials of 472 hospitalized children and 281 children attending day care centers, *Lactobacillus GG* administration caused a decrease in the risk of respiratory tract infections (92, 93). A recent meta-analysis showed an insignificant association of probiotic use with the reduction in asthma risk; emerging from these studies, was a heterogeneity in the type of probiotics used and quality of bacterial identification technique (94–96). However, in other two meta-analyses from 2016 and 2015, a decrease in the respiratory tract infection rate valuated on the number of new episodes and number of days of fever was demonstrated despite the heterogeneity of the probiotics administration (97, 98). Table 2 summarizes the clinical trials evaluating probiotic administration.

**Table 2:** Probiotic supplementary therapy explored in randomized controlled trials.

**Type of study**	**Structure**	**Subjects**	**Results**	**Limitations or subgroup analysis**
Meta-analysis (89)	Four randomized, placebo-controlled trials involving *Lactobacillus Rhamnosus* GG supplementation	1,805 participants	*Lactobacillus Rhamnosus* GG administration was associated with a reduced incidence of acute otitis media, and of upper respiratory infections	There was no significant difference between the *Lactobacillus Rhamnosus* GG and the control groups in the risk of overall respiratory infections
Double-blind, prospective, randomized, placebo-controlled trial (91)	Patients randomized to receive *Lactobacillus paracasei* (LP), *Lactobacillus fermentum* (LF), LP + LF, or a placebo for 3 months	160 children with asthma aged 6–18 years	The LP + LF group demonstrated increased peak expiratory flow rates and decreased IgE levels	
Meta-analysis (94)	17 randomized controlled trials	5,264 children	Probiotic use do not reduce the asthma risk	Effects were based on the type of probiotics used, which also need more large-sample and high-quality studies
Meta-analysis (95)	19 randomized controlled trials	5,157 children	Probiotic supplementation compared with placebo was not associated with a lower risk of asthma in infant	
Systematic review (96)	14 randomized controlled trials		Probiotics in immunocompetent children have a modest effect both in diminishing the incidence of upper respiratory infections and the severity of the infection symptoms	
Meta-analysis (97)	23 randomized trials	6,269 children	Probiotics significantly decreased the number of subjects having at least 1 respiratory tract infection episode.	
Systematic review (98)	11 randomized clinical trials	2,417 children	Reduction in new episodes of disease was a favorable outcome for the use of probiotics in the treatment of respiratory infections	Studies showed to be heterogeneous regarding strains of probiotics, the mode of administration, the time of use, and outcomes

There is a lack of favorable evidence for probiotic use in preventing subsequent asthma or allergy because the complexity of the microbiome–human interaction is greater than a simple cause-and-effect relationship of probiotic administration. The influence of diet in late adolescence on the microbiome composition with its consequent influences in lung also seems to be an important primary preventive factor for allergic disease (99). The use of probiotics with live microorganisms for preventing or curing respiratory infections has not been clearly defined, and there are no scientific recommendations about the use of probiotics because the quality of evidence is low, but there are some trials that seem to provide important positive evidence for a future clinical scientific approach of probiotic use.

## Conclusion

A strong relationship exists between the lung and intestinal microbiota, the environment, and the effects of early life exposure to non-pathogenic microbes of the natural environment, which are important for immune system development. The axis between the gut and lung is important for immune tolerance, which can determine the susceptibility to developing asthma or allergy, in particular, during the early phases of immune system structuring. Despite important correlations between microbiota and inflammation or immune response homeostasis in preclinical studies and in specific group of patients, there is not enough robust evidence, except for a few efficacious results from randomized clinical trials, which are heterogeneous for probiotics dose and sampling analysis, to recommend the general use of probiotics to prevent asthma or allergy. Further studies are needed to better understand the role of the microbiota in respiratory diseases and to define the possibility of a therapeutic intervention with probiotics or prebiotics or simply, an early life with more rural experiences.

## Author Contributions

AL developed the original idea and the final revision. GP and GFP wrote the manuscript. AG, SM, and MP revised firstly the manuscript and contributed to English revision and references update. SS, GM, and SL made the final analysis and critical revision of the manuscript. All authors read and approved the final manuscript.

### Conflict of Interest

The authors declare that the research was conducted in the absence of any commercial or financial relationships that could be construed as a potential conflict of interest.
